# Association of migraine with subsequent risk of depression or anxiety by small-area deprivation: national cohort study with sibling analysis

**DOI:** 10.1186/s10194-026-02280-8

**Published:** 2026-02-23

**Authors:** Emily White Johansson, Mattias Linde, Anna Ohlis, Mathias Mattsson, Ahmed Nabil Shaaban, Sofie Gustafsson, Johan Holm, Christina Dalman, Emilie E. Agardh

**Affiliations:** 1 Department of Global Public Health, Karolinska Institutet, Stockholm, Sweden; 2 Global Health and Migration Unit, Department of Women’s and Children’s Health, Uppsala University, Akademiska Sjukhuset, Uppsala, 751 85 Sweden; 3 Department of Neuromedicine and Movement Science, Norwegian University of Science and Technology (NTNU), Trondheim, Norway; 4 Regional Migraine Unit, Sahlgrenska University Hospital, Gothenburg, Sweden; 5 Centre for Epidemiology and Community Medicine, Region Stockholm, Stockholm, Sweden; 6 Pfizer AB, Stockholm, Sweden

**Keywords:** Migraine, Depression, Anxiety, Residential deprivation, Sweden

## Abstract

**Background:**

Growing evidence indicates that migraine could increase the risk of depression or anxiety in affected persons. Residential deprivation, with its links to stressful environments and access to quality services, could exacerbate the risk of developing depression or anxiety among migraine patients, but evidence remains limited. We aimed to examine the subsequent risk of depression or anxiety in persons with migraine and to determine if this association varies by small-area deprivation.

**Methods:**

A nationwide register-based cohort study was conducted of persons aged 10–50 years registered in Sweden from 2015 to 2023 who did not have recorded depression or anxiety in the previous ten years (*n* = 4,065,375). We generated matched (*n* = 1,455,352) and sibling (*n* = 179,888) samples for analyses. The exposure (migraine) and outcome (depression or anxiety) were defined as the first diagnosis, or the first prescription date if no diagnosis was recorded in the study period. Follow-up started six months after migraine exposure and hazard ratios (HR) for depression or anxiety through 31 December 2023 were computed for the matched and sibling cohorts using stratified cox regression models with robust error variance. We tested for an interaction between small-area deprivation and migraine on the association with depression or anxiety.

**Results:**

Incidence rates for depression or anxiety among people with or without migraine in the matched cohort were 39.2 and 21.9 per 1,000 person-years with an adjusted HR of 1.78 (95% CI: 1.75–1.81) across follow-up. In the sibling cohort, the HR of depression or anxiety associated with migraine was 1.63 (95% CI: 1.58–1.68). There was no evidence of an overall interaction between migraine and small-area deprivation on the subsequent risk of depression or anxiety in the matched (*p* = 0.123) or sibling (*p* = 0.422) cohorts.

**Conclusions:**

People with migraine have a nearly two-fold higher rate of depression or anxiety than those without migraine, and the association was lower but remained after adjustment for shared genetics or childhood family environments. While small-area deprivation did not modify the relationship between migraine and the risk of depression or anxiety in our cohort, further investigation of this important research question is merited.

**Supplementary Information:**

The online version contains supplementary material available at 10.1186/s10194-026-02280-8.

## Introduction

Migraine is the second leading cause of disability worldwide and affects more than one billion people annually [1]. In Sweden, it has been estimated that at least one in eight individuals experience migraine with women and people in lower socioeconomic groups disproportionately affected [2–4]. Migraine not only affects a person’s health and functioning, but may also lead to disruptions in social relationships, leisure time, school activities, ability to work, and income [5–7]. Furthermore, migraine is often co-morbid with depression or anxiety, further worsening health and wellbeing, working life, and relationships [8–15]. Similar to migraine, depression and anxiety are two major public health problems that often onset in adolescence or early adulthood and are more common in people with lower education or income levels [9, 16, 17].

A growing body of evidence shows the relationship between migraine, depression, and anxiety could be bidirectional with the conditions potentially exacerbating one another [8, 12, 15, 18]. Migraine may increase the risk of depression and anxiety, suggesting that severe pain could potentially lower a person’s quality of life leading to depressive symptoms [8, 12, 13]. There is also potential that the association between migraine and development of depression or anxiety is exacerbated by other known risk factors [9, 13, 16, 19–22], such as genetics or environmental conditions, but evidence remains limited.

Residential deprivation has long been established as an important driver of poor health outcomes including depression and anxiety [19, 23–27]. Residential deprivation refers to neighborhoods that are socioeconomically disadvantaged across multiple domains such as housing, employment, services, safety, or other resources [19, 23–27], which is referred to in this paper as small-area deprivation. Such deprivations are linked to stress levels, crime and violence, access to quality services, social cohesion, among other mechanisms that are known to influence depression or anxiety risk [23–25]. Yet evidence is lacking on whether small-area deprivation plays a modifying role in the potential bidirectional relationships between migraine, depression, and anxiety.

To help fill this evidence gap, this study aims to examine if people with migraine have increased subsequent risk of depression or anxiety and if the association differs according to small-area deprivation.

## Methods

We conducted a national register-based cohort study with matched and sibling samples to examine the association between migraine and depression or anxiety among persons aged 10–50 years registered in Sweden between 2015 and 2023. The role of small-area deprivation was studied as a potential modifier in the risk of depression or anxiety among persons with migraine.

### Data sources

Using a Swedish personal identification number uniquely assigned to each resident, the study linked multiple healthcare registers at the national and regional levels, including: (1) the Total Population Register for population and household statistics; (2) the National Prescribed Drug Register for prescription medications dispensed through Swedish pharmacies; (3) the National Multigenerational Register: and (5) the National Swedish Longitudinal Database for Health Insurance and Labor Market Studies for socioeconomic information for adult residents in Sweden. (6) the National Patient Register for data on the management of patients seen through inpatient and specialized outpatient care in Swedish hospitals; and (3) the Primary Healthcare Registers for data on the management of patients at regional primary healthcare centers. All registers had national coverage for the study period except the primary care registers that were available for 17 of 21 regions in the years 2015–2017, representing 89% of the Swedish population. The study received approval from the Ethical Review Authority in Sweden (2018/1339-31/5, 2018/2292-32, 2019–02185, 2021 − 00657, 2022-03111-02, 2023-07509-02, 2024-02816-02).

### Study population

**Fig. 1:**
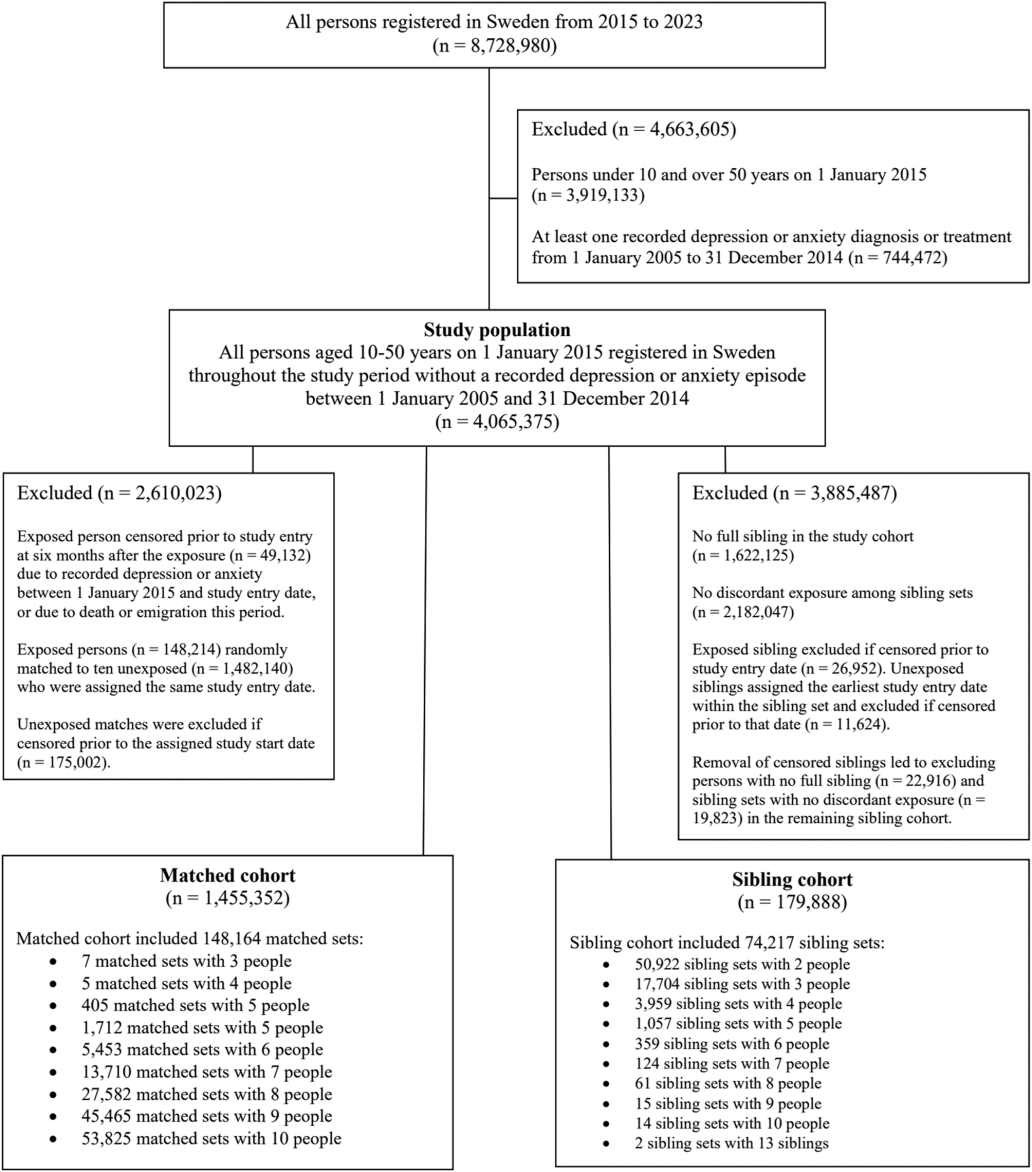
Flow diagram of inclusion criteria for the study population and sampled cohorts

We identified all persons registered in Sweden throughout the study period from 2015 to 2023 who had a personal identification number assigned at birth or immigration, and a primary address assigned to one of 5,984 small geographical divisions established by Statistics Sweden (DeSO) (Fig. 1) (*n* = 8,728,980) [28, 29]. We restricted the study cohort to people aged 10–50 years (*n* = 3,919,133) since migraine onset typically occurs within this age range and there is broad homogeneity in the pathophysiology and management of the three conditions within this age range [8]. We further excluded 744,472 people diagnosed with or treated for depression or anxiety between 1 January 2005 and 31 December 2014, or ten years prior to the study start on 1 January 2015 (Table S1-S2). This was done to further establish a temporal relationship between exposure and outcome in our analysis. The final study population included 4,065,375 individuals.

### Matched and sibling cohorts

From the study population, we generated separate matched and sibling samples for analysis purposes. The age- and sex- matched cohort was generated by identifying the 197,346 exposed person in the study population and excluding those who were censored prior to entering follow-up at six months after their first recorded migraine (*n* = 49,132). Persons were censored prior to study entry if they had recorded depression or anxiety between 1 January 2015 and their study entry date, or who died or emigrated during this time. A total of 148,214 exposed persons were randomly matched to ten unexposed based on sex and five-year age groups (*n* = 1,482,140). All persons in each matched set were assigned the same study entry date, which was six months after the first recorded migraine date for the exposed person in the matched set. We excluded any unexposed matches who were censored prior to the follow-up start date for the reasons previously described (*n* = 175,002). The final sample for the matched cohort was 1,455,352 persons including 148,164 matched sets.

The sibling cohort was separately generated by identifying full siblings in the study population who had the same mother and father in the Swedish Multigeneration Register. We excluded people with no full sibling (*n* = 1,622,125) and sibling sets with no discordant exposures (*n* = 2,182,047). As per the matched cohort procedure, we excluded exposed siblings who were censored prior to entering follow-up at six months after their first recorded migraine exposure (*n* = 26,952). Unexposed siblings were assigned the earliest exposure date within the sibling set and excluded if censored prior to this study entry date (*n* = 11,624). The removal of censored siblings led to excluding persons in the remaining cohort with no full sibling (*n* = 22,916) and sibling sets with no discordant exposure (*n* = 19,823). The final sample for the sibling cohort was 179,888 persons including 74,217 sibling sets.

### Migraine measurement

Migraine was defined as the first diagnosis date in primary, specialist outpatient or inpatient care, and if no diagnosis was recorded during the study period, the first prescription date for a dispensed drug. Table S3 lists the diagnosis and prescription codes that measured the exposure based on the Swedish adaptation of the International Statistical Classification of Diseases and Related Health Problems, 10th version (ICD-10-SE) and the Anatomical Therapeutic Chemical (ATC) classification system, respectively. Migraine diagnoses were derived from any inpatient admission or specialist outpatient visit recorded in the National Patient Register during the study period, and any primary care visit in 17 of 21 regions from 2015 to 2017 when data were available. If the date of diagnosis (day and month) was missing, we imputed the date using the earliest prescription date in the year of the visit. If there was no prescription that year, the midpoint date in the year of the visit was used for the diagnosis date. Study participants without recorded migraine as previously described comprised the comparison group.

### Depression or anxiety measurement

Depression or anxiety was defined as the first diagnosis date in primary, specialist outpatient or inpatient care, and if no diagnosis was recorded in the study period, the first prescription date for a dispensed drug. Table S2 lists the ICD-10-SE and ATC codes that measured the outcome in administrative registers. Depression or anxiety diagnoses were derived from any inpatient admission or specialist outpatient visit during the study period, and any primary care visit in 17 of 21 regions from 2015 to 2017 when data were available. If the diagnosis date was missing (day and month), we imputed the date using the earliest prescription date in the year of the visit. If there was no prescription in that year, the midpoint date in the year of the visit was used for the diagnosis date.

### Small-area deprivation level

We used the Index for Multiple Deprivation in Sweden (IMDIS) to estimate small-area deprivation level for each of the 5,984 DeSO areas in the year 2015 [30]. IMDIS has been previously described elsewhere [30]. Briefly, IMDIS is a composite index of deprivation that aims to measure four domains using 15 indicators: (1) income and capital (2) education (3) employment (4) housing. An IMDIS valued based on this composite measure was assigned to each DeSO, which was then ranked from least to most deprived and categorized into quartiles using the 25th, 50th, and 75th percentiles (very low, low, high, and very high deprivation levels). Each person was assigned the deprivation level of the DeSO for their primary address in the Total Population Register in the year of their study entry. We assumed the deprivation level assigned to each DeSO in the year 2015 remain unchanged across the study period that could lead to exposure misclassification if residential area characteristics changed over time. Previous research has shown that nearly 80% of Stockholm neighborhoods had stable socioeconomic profiles from 1990 to 2015 [31]. While this research does not cover all of Sweden or the most recent period from 2015 onwards, it does suggest that the vast majority of the nearly 6,000 small geographic areas in our analysis could likely be classified into the same deprivation quartile during the study period.

### Other covariates

Other regression covariates included age in the year of study entry (continuous), sex (male or female), area of residence in the year of study entry (urban, peri-urban, rural), and birthplace (Sweden, Nordic outside Sweden, European Union (EU28) outside Nordic, Europe outside EU28 and Nordic, or another birthplace) derived from the Total Population Register. Area of residence was defined by Statistics Sweden and derived from the DeSO assigned to each study participant.

### Statistical analyses

For matched and sibling cohort analyses, we separately calculated unadjusted rates of depression or anxiety per 1,000 person-years for participants with or without migraine. Person-years at risk were computed from the start of follow-up until date of outcome, death, emigration, or study end on 31 December 2023, whichever occurred first. Cumulative incidence rates for depression or anxiety among participants with or without migraine were plotted using the Kaplan-Meier method and compared using the log rank test.

Stratified cox proportional hazards models with robust error variance and separate strata for each sibling or matched set were used to compute hazard ratios (HR) with 95% confidence intervals (CI) for the association between migraine and the outcome, using no migraine as the reference category. We tested the proportional hazards assumption by inspecting the log minus log survival plot in matched and sibling cohorts (Figures S1-S2). Final models were adjusted for small-area deprivation level, age, sex, area of residence, and birthplace. We used a Wald Test to determine if there was an interaction between migraine and small-area deprivation level in the adjusted models of the matched and sibling cohorts. The level of statistical significance was set to 0.05. We analyzed data using Stata 18 (StataCorp, College Station, TX).

### Sensitivity analyses

We conducted two sensitivity analyses for this study. In the first sensitivity analysis the definition of the outcome was restricted to diagnoses only, and prescribed depression or anxiety drugs (without a diagnosis) were omitted from the definition since some antidepressants may be used to treat other conditions, such as chronic pain. In the second sensitivity analysis, the outcome was defined without primary healthcare data since these data were not available throughout the study period or for all regions.

## Results

Among the 4,065,375 participants, 4.9% had recorded migraine between 2015 and 2023 (Table 1). There was limited variation in this proportion across small-area deprivation levels. The median age of participants with migraine was 30 years (IQR: 21–41) and three times as many women had migraine than men in the study population. The matched sample had a similar exposure distribution to the study population across all covariates except those used for matching. The sibling sample, in comparison to the study population, had a higher proportion of men, native-born Swedes, rural and peri-urban residents, and residents in less deprived areas.

**Table 1:** Characteristics of the study population and sampled cohorts by migraine status

	Study population					Matched cohort					Sibling cohort			
	Migraine		No Migraine			Migraine		No Migraine			Migraine		No Migraine	
	*N*	%	*N*	%		*N*	%	*N*	%		*N*	%	*N*	%
Total	197 346	100.0	3 868 029	100.0		148 214	100.0	1 307 138	100.0		76 239	100.0	103 649	100.0
**Age**,** median (IQR)**	30	(21–41)	30	(20–41)		34	(25–44)	34	(24–44)		32	(23–42)	31	(23–42)
**Sex**														
Male	52 847	26.8	2 116 778	54.7		43 278	29.2	401 237	30.7		22 243	29.2	60 009	57.9
Female	144 499	73.2	1 751 251	45.3		104 936	70.8	905 901	69.3		53 996	70.8	43 640	42.1
**Small-area deprivation**														
Very low deprivation	50 950	25.8	1 004 129	26.0		39 225	26.5	338 601	25.9		21 526	28.2	26 779	25.8
Low deprivation	50 419	25.6	984 068	25.4		37 867	25.6	331 780	25.4		20 689	27.1	27 167	26.2
High deprivation	48 799	24.7	962 372	24.9		36 068	24.3	324 110	24.8		18 792	24.7	26 301	25.4
Very high deprivation	47 178	23.9	917 460	23.7		35 054	23.7	312 647	23.9		15 232	20.0	23 402	22.6
**Area of residence**														
Urban	153 938	78.0	2 965 186	76.7		115 335	77.8	1 010 357	77.3		57 922	75.2	77 976	76.0
Peri-urban	16 550	8.4	329 694	8.5		12 467	8.4	108 386	8.3		6 834	8.9	9 225	9.0
Rural	26 858	13.6	573 149	14.8		20 412	13.8	188 395	14.4		11 483	15.9	16 448	15.1
**Birthplace**														
Sweden	159 913	81.3	3 144 715	81.3		119 929	80.9	1 048 927	80.3		71 066	93.2	94 228	90.9
Nordic outside Sweden	2 309	1.1	43 278	1.1		1 792	1.2	15 542	1.2		354	0.5	489	0.5
EU28 outside Nordic	5 763	3.4	130 001	3.4		4 309	2.9	45 554	3.5		487	0.6	710	0.7
Europe outside EU28	6 113	2.8	109 697	2.8		4 515	3.1	38 311	2.9		1 178	1.6	2 066	2.0
Other birthplace	23 248	11.4	440 338	11.4		17 669	11.9	158 804	12.2		3 154	4.1	6 156	5.9

### Matched analysis

**Fig. 2:**
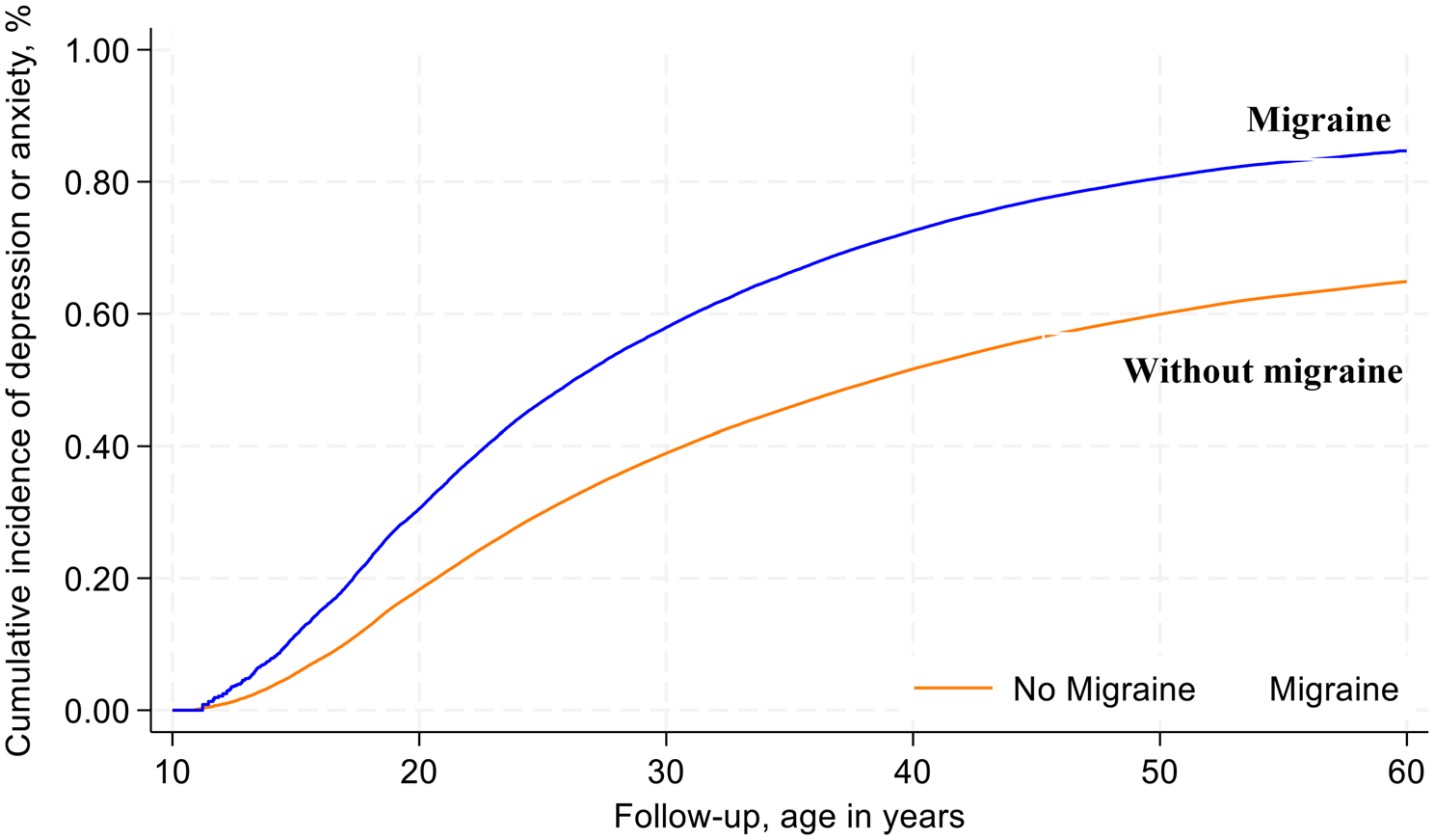
Kaplan-Meier cumulative incidence rates of depression or anxiety through 31 December 2023 among the matched cohort

During 7,214,949 person-years of follow-up, a total of 169,733 individuals had a depression or anxiety episode (median time at risk: 5.4 years) (Table 2). Among participants with the outcome, 26,521 had migraine corresponding to an incidence rate of 39.2 episodes per 1,000 person-years (95% CI: 38.7–39.6) compared to 21.9 per 1,000 person-years (95% CI: 21.8–22.0) for people without migraine. Cumulative incidence curves on the matched cohort showed consistently higher probability of developing depression or anxiety among people with migraine versus those without migraine across the study duration (Fig. 2). Overall, migraine was associated with a 1.78 (95% CI: 1.75–1.81) higher rate of depression or anxiety compared to no migraine in adjusted models across the study period (Table 2).

### Sibling analysis

The sibling analysis included 862,613 person-years of follow-up with a total of 23,612 full siblings experiencing depression or anxiety across the follow-up period (Table 2). Among siblings with depression or anxiety, 13,250 siblings had migraine corresponding to an incidence rate of 37.7 episodes per 1,000 person-years (95% CI: 37.0-38.3) compared to 20.3 per 1,000 person-years (95% CI: 19.9–20.7) for siblings without migraine. In the sibling analysis, migraine was associated with a 1.63 (95% CI: 1.58–1.68) higher rate of depression or anxiety compared to no migraine in adjusted models across the study period.

**Table 2:** Association between migraine and depression or anxiety through end-2023 in the matched and sibling cohorts

	Persons entering follow-up	Depression or anxiety episodes	Person-years at risk	Rate of depression or anxiety episodes	Hazard ratios (95% CI)			
	*N*	*N*	*N*	per 1,000 person-years	Crude	*p*-value	Adjusted	*p*-value
**Matched sample**	**1 455 352**	**169 733**	**7 214 949**					
Person with migraine	148 214	26 521	677 418	39.2 (38.7–39.6)	1.78 (1.76–1.81)	< 0.001	1.78 (1.75–1.81)	< 0.001
Person with no migraine	1 307 138	143 212	6 537 531	21.9 (21.8–22.0)	1.00		1.00	
**Sibling sample**	**179 888**	**23 612**	**862 613**					
Sibling with migraine	76 239	13 250	351 732	37.7 (37.0–38.3)	1.89 (1.83–1.94)	< 0.001	1.63 (1.58–1.68)	< 0.001
Sibling with no migraine	103 649	10 362	510 881	20.3 (19.9–20.7)	1.00		1.00	

Note: Hazard ratios were estimated using stratified Cox regression models with strata for each matched or sibling set and robust error variance. Adjusted hazard ratios accounted for age, sex, small-area deprivation, area of residence, and birthplace. For the matched cohort, persons with migraine were matched to ten unexposed by sex and five-year age-bands. For the sibling cohort, siblings with migraine were matched to unexposed siblings

### Interaction between small-area deprivation and migraine on risk of depression or anxiety

**Fig. 3:**
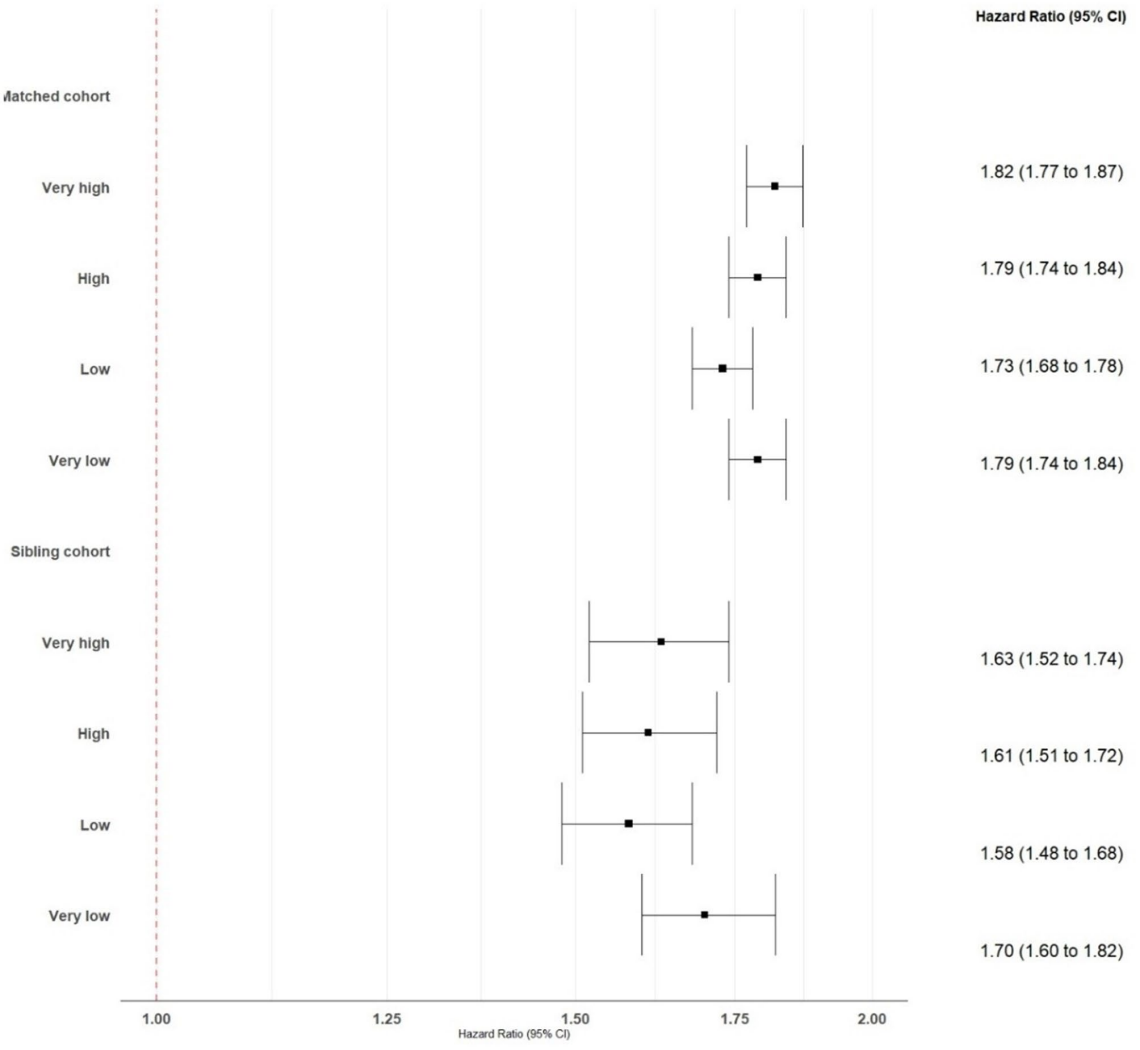
Associations between migraine and depression or anxiety through end-2023 in the matched and sibling cohorts of persons aged 10–50 years registered in Sweden in 2015–2023, by small-area deprivation

There was no overall evidence of an interaction between migraine and small-area deprivation level on the subsequent risk of depression or anxiety in the matched (*p* = 0.123) or sibling (*p* = 0.422) cohorts. In the matched cohort, the interaction term between low and very high deprivation levels was not significant (*p* = 0.089) although the CIs across these deprivation levels had limited overlap (Fig. 3).

### Sensitivity analysis

In the sensitivity analysis that used a stricter definition without drug exposure for depression and anxiety (Table S4), there were 7,565,649 person-years of follow-up in the matched cohort and a total of 83,418 people diagnosed with the outcome. After adjustment, the HR for depression or anxiety associated with migraine was 1.73 (95% CI: 1.70–1.76) across the study duration. In the sensitivity analysis that did not include primary healthcare data, an adjusted HR for depression or anxiety associated with migraine of 1.96 (95% CI: 1.93–1.99) was found in the matched cohort (Table S5).

## Discussion

### Principle findings

In this national register-based cohort study with matched and sibling analyses, we found that people who were depression- or anxiety-free for the ten years prior to their migraine diagnosis had a higher subsequent risk of these conditions compared to people without migraine. This association was lower but remained significant in the sibling analysis that further adjusted for shared genetics and childhood family environments. We found no evidence for the modifying role of small-area deprivation in the development of depression or anxiety among migraine patients in our cohort. This important research area merits further investigation, particularly in settings with larger socioeconomic disparities across residential areas.

### Comparisons with other studies

The comorbidity of depression, anxiety and migraine symptoms is well-documented [8, 9, 11–13, 15, 32], although the reason for this overlap is less well understood [8, 13]. A growing body of evidence suggests a bidirectional relationship between the three conditions with one or more conditions inducing the other(s) [18, 33]. Recent research has shown that people with migraine had increased risks of affective or anxiety disorders [8, 33, 34].

Our study expanded on previous research by analyzing matched and sibling samples drawn from a national cohort to examine the associated subsequent risk of depression or anxiety among migraine patients with no recent history of these conditions. We found a nearly two-fold higher rate of depression or anxiety among people with migraine in the matched cohort. When accounting for shared childhood family environment and genetic factors, which are known risk factors for all three conditions, the association remained but was slightly weaker in the sibling analysis.

Building on this evidence, our study is the first to our knowledge to examine if living in a highly deprived area exacerbates the subsequent risk of depression or anxiety in migraine patients. Residential deprivation itself has been shown to increase the risk of depression or anxiety in previous research from Sweden and elsewhere [24, 26], yet there has been limited evidence of its potential modifying role in any bidirectional relationship. It is plausible that a bidirectional relationship between migraine with depression or anxiety could be influenced by residential deprivation given its known links to stress, crime and violence, access the quality services, social cohesion, among other environmental factors [35–38]. Yet, we found no overall role of small-area deprivation in modifying the relationship between migraine and the subsequent risk of depression or anxiety in our cohort. Our analysis tested multiplicative interactions on the hazard ratio scale, and this does not exclude absolute risk differences between deprivation quartiles. It is also possible that our matched and sibling cohorts did not have sufficient power to detect effect differences across deprivation levels, or that persons excluded from the study population with prior depression or anxiety were correlated with greater residential deprivation further obscuring these results. In addition, a separate cross-sectional analysis of this study population showed higher migraine prevalence in residents of the least-deprived areas [39], potentially due to having more health system contacts for migraine diagnosis and treatment. In contrast, previous research suggests an opposite relationship for small-area deprivation and depression or anxiety prevalence [22, 24]. Taken together, these results suggest a more complicated role for small-area deprivation in the development of these conditions and potentially in terms of modifying any potential bidirectional relationship. Further research is needed to better elucidate to what extent, if any, social and environmental conditions may modify how these conditions relate to one another.

### Strengths and limitations of this study

This study leveraged multiple national and regional registers to enable research on a whole population basis providing greater statistical power for analyzing matched and sibling cohorts, stratifying by small-area deprivation level, minimizing risk of selection bias, and allowing for a longer and more complete follow-up period. There are also some study limitations. First, the study relied on administrative registers to ascertain exposure and outcome status in the population that may undercount and misclassify cases due to well-recognized gaps in care-seeking and appropriate management of these conditions. Misclassification of migraine, depression and anxiety from register data, including under-ascertainment, may disproportionately affect men and milder cases. In addition, regional primary care registers were only available for 17 of 21 regions (89% national population) and for the years 2015 to 2017. This will likely underestimate the prevalence of all three conditions in the current study and could potentially impact associations found if the three conditions are differentially diagnosed in primary versus specialist or inpatient care where measurement is at the national level, or if drugs are prescribed in ways that are systematically different across the conditions. A large proportion of cases were ascertained from prescription registers alone. We included only migraine drugs that did not have multiple indications, and the registers do not include over- the-counter drugs used to treat migraine. In contrast, antidepressants could be prescribed for other conditions, e.g., chronic pain, and we aimed to account for this issue in sensitivity analysis using diagnosed cases only. Second, observational studies are prone to residual confounding that makes causal inferences difficult. Migraine may not directly cause depression or anxiety, and the associations found in our study may be due to unmeasured risk factors associated with both the exposure and outcome. For example, people with migraines may have more contact with the healthcare system than undiagnosed people and could thus be more likely to get diagnosed with other conditions such as depression or anxiety. The sibling analysis could partially adjust for healthcare seeking behaviors learned in a shared childhood family environment, but residual confounding likely remains. Fourth, while sibling analyses may reduce confounding bias from shared genetic and other unmeasured factors in the childhood family environment, sibling comparisons are prone to carryover effects and may also lead to different associations from the matched comparison due to poor generalizability and varying distributions of other confounders in the sibling versus matched samples. Sibling samples may also undercount immigrant families with siblings who may not be resident in Sweden or sought healthcare here. Fifth, contextual factors such as small-area deprivation have measurement challenges including defining the area or developing indicators, and composite deprivation measures could obscure differences in areas even within the same level. Sixth, our study excluded people with depression or anxiety in the previous ten years, which may introduce selection or collider bias and change the demographic composition of our study cohort compared to the general Swedish population. This bias could attenuate both the overall association between migraine and the outcome as well as the interaction by small-area deprivation if depression or anxiety is correlated with living in more deprived areas. It is also possible that persons may be misclassified as having no prior mental health conditions if they did not seek care or were treated outside Sweden in the past ten years. Finally, small-area deprivation level for each DeSO was measured in 2015 and the quartile grouping was assumed to remain unchanged throughout the study period.

## Conclusions

People with migraine have a higher subsequent risk of depression or anxiety than those without migraine, and this association remained but was lower in sibling analyses after adjusting for shared genetic factors and childhood family environments. We found no evidence of a modifying role for small-area deprivation in the subsequent risk of depression or anxiety among migraine patients, although further investigation is merited.

## Supplementary Information

Below is the link to the electronic supplementary material.

Supplementary Material 1

## Data Availability

Due to ethical restrictions, the individual level data used in this study are not publicly available. However, the data supporting the findings of this study can be obtained from Statistics Sweden (www.scb.se) and the Swedish National Board of Health and Welfare (www.socialstyrelsen.se).
