# Supplementary material for: Association of migraine with subsequent risk of depression or anxiety by small-area deprivation: national cohort study with sibling analysis
Source: J Headache Pain. 2026 Feb 23;27(1):57. doi: 10.1186/s10194-026-02280-8 (PMC12930664; doi:10.1186/s10194-026-02280-8)
Supplement: Supplementary file 1 — Supplementary Material 1 [file 10194_2026_2280_MOESM1_ESM.docx]

| **Title** | **Role of small-area deprivation in the risk of depression or anxiety among people with migraine: national cohort study with sibling analysis**  **SUPPLEMENTARY MATERIALS** |
| --- | --- |
| **Author names** | Emily White Johansson^1, 2^  Mattias Linde ^3, 4^  Anna Ohlis ^1,5^  Mathias Mattson^1^  Ahmed Nabil Shaaban^1^  Sofie Gustafsson^1, 6^  Johan Holm^6^  Christina Dalman^1^  Emilie E Agardh^1^ |
| **Author information** | 1. Department of Global Public Health, Karolinska Institutet, Stockholm, Sweden 2. Global Health and Migration Unit, Department of Women’s and Children’s Health, Uppsala University, Uppsala, Sweden 3. Department of Neuromedicine and Movement Science, Norwegian University of Science and Technology (NTNU), Trondheim, Norway 4. Regional Migraine Unit, Sahlgrenska University Hospital, Gothenburg, Sweden 5. Centre for Epidemiology and Community Medicine, Region Stockholm, Stockholm, Sweden 6. Pfizer AB, Stockholm, Sweden |
| **Corresponding author** | Emily White Johansson PhD  Associate Professor  Uppsala University  Department of Women’s and Children’s Health  Global Health and Migration Unit  Akademiska sjukhuset  751 85 Uppsala, Sweden  Email: [emily.johansson@uu.se](mailto:emily.johansson@uu.se)  ORCiD ID: 0000-0001-5162-8277  Karolinska Institutet  Department of Global Public Health |

**Supplementary materials**

Table S1: Characteristics of persons excluded from the study population due to prior depression or anxiety in the ten years prior to the study start date

Table S2: ICD-10-SE and ATC codes for depression or anxiety measurement

Table S3: ICD-10-SE and ATC codes for migraine measurement

Table S4: Sensitivity analysis of the association between migraine and diagnosed depression or anxiety through end-2023 in the matched cohort of persons aged 10-50 years registered in Sweden from 2015 to 2023

Figure S1: Log minus log survival plot of depression or anxiety through end-2023 in the matched cohort

Figure S2: Log minus log survival plot of depression or anxiety through end-2023 in the sibling cohort

Table S5: Sensitivity analysis of the association between migraine and depression or anxiety through end-2023 in the matched cohort of persons aged 10-50 years registered in Sweden from 2015 to 2023 (excluding primary healthcare data)

Table S1: Characteristics of persons excluded from the study population due to prior depression or anxiety in the ten years prior to the study start date

|  | **Persons excluded from the study population due to prior depression or anxiety** | | | |
| --- | --- | --- | --- | --- |
|  | Migraine | | No Migraine | |
|  | N | % | N | % |
| **Total** | **78 132** | **100.0** | **666 340** | **100.0** |
| **Age, median (IQR)** | 33 | (23, 42) | 31 | (22, 42) |
| **Sex** |  |  |  |  |
| Male | 12 829 | 16.4 | 265 074 | 39.8 |
| Female | 65 303 | 83.6 | 401 266 | 60.2 |
| **Small-area deprivation** |  |  |  |  |
| Very low deprivation | 17 011 | 21.8 | 144 025 | 21.6 |
| Low deprivation | 19 663 | 25.2 | 166 946 | 25.1 |
| High deprivation | 20 259 | 25.9 | 172 918 | 26.0 |
| Very high deprivation | 21 199 | 27.1 | 182 451 | 27.4 |
| **Area of residence** |  |  |  |  |
| Urban | 61 236 | 78.4 | 524 386 | 78.7 |
| Peri-urban | 6 578 | 8.4 | 54 376 | 8.2 |
| Rural | 10 318 | 13.2 | 87 578 | 13.1 |
| **Birthplace** |  |  |  |  |
| Sweden | 64 496 | 82.5 | 556 992 | 83.6 |
| Nordic outside Sweden | 1 099 | 1.4 | 9 097 | 1.4 |
| EU28 outside Nordic | 1 835 | 2.3 | 15 364 | 2.3 |
| Europe outside EU28 & Nordic | 2 619 | 3.4 | 22 565 | 3.4 |
| Other birthplace | 8 083 | 10.3 | 62 322 | 9.4 |

Table S2: ICD-10-SE and ATC codes for depression or anxiety measurement

| **Depression or anxiety outcome measurement** | | |
| --- | --- | --- |
| **Diagnosis (ICD-10-SE) codes** | | |
| Depressive episode | F32 | Includes all subcategories |
| Major depressive disorder, recurrent | F33 | Includes all subcategories |
| Affective disorders | F34.1 | Dysthymic disorder |
| Phobic anxiety disorders | F40 | Includes all subcategories |
| Other anxiety disorders | F41 | Includes all subcategories |
| Reaction to severe stress and adjustment disorders | F43 | Includes all subcategories |
| Dissociative and conversion disorders | F44 | Includes all subcategories |
| Emotional disorders with onset specific to childhood | F93.0 | Separation anxiety disorder in childhood |
|  | F93.1 | Phobic anxiety in childhood |
|  | F93.2 | Social anxiety in childhood |
| **Prescription (ATC) codes** | | |
| SSRI | N06AB01 | Viloxazin |
| SSRI | N06AB02 | Zimelidin |
| SSRI | N06AB03 | Fluoxetin |
| SSRI | N06AB04 | Citalopram |
| SSRI | N06AB05 | Paroxetin |
| SSRI | N06AB06 | Sertralin |
| SSRI | N06AB07 | Alaproklat |
| SSRI | N06AB08 | Fluvoxamin |
| SSRI | N06AB09 | Etoperidon |
| SSRI | N06AB10 | Escitalopram |
| Tetracyclic antidepressants | N06AC03 | Mirtazapin |
| Modified cyclic antidepressants | N06AD01 | Nomifensin |
| Monocyclic antidepressants | N06AE02 | Fluvoxamin |
| Monocyclic antidepressants | N06AE06 | Venlafaxin |
| MAO inhibitors, non-selective | N06AF01 | Isokarboxazid |
| MAO inhibitors, non-selective | N06AF02 | Nialamid |
| MAO inhibitors, non-selective | N06AF03 | Fenelzin |
| MAO inhibitors, non-selective | [N06AF04](https://www.fass.se/LIF/atcregister?1-1.-atccontainer-atcnavigatepanel4-listcontainer-atccodelistview-3-atclink&userType=0&atcCode=N06A) | Tranylcypromin |
| MAO inhibitors, non-selective | N06AF05 | Iproniazid |
| MAO inhibitors, non-selective | N06AF06 | Iproklozid |
| Other antidepressants | N06AX01 | Oxitriptan |
| Other antidepressants | N06AX04 | Nomifensin |
| Other antidepressants | N06AX05 | Trazodon |
| Other antidepressants | N06AX06 | Nefazodon |
| Other antidepressants | N06AX07 | Minaprin |
| Other antidepressants | N06AX08 | Bifemelan |
| Other antidepressants | N06AX09 | Viloxazin |
| Other antidepressants | N06AX10 | Oxaflozan |
| Other antidepressants | [N06AX11](https://www.fass.se/LIF/atcregister?1-1.-atccontainer-atcnavigatepanel4-listcontainer-atccodelistview-10-atclink&userType=0&atcCode=N06A) | Mirtazapin |
| Other antidepressants | N06AX13 | Medifoxamin |
| Other antidepressants | N06AX14 | Tianeptin |
| Other antidepressants | N06AX15 | Pivagabin |
| Other antidepressants | [N06AX16](https://www.fass.se/LIF/atcregister?1-1.-atccontainer-atcnavigatepanel4-listcontainer-atccodelistview-15-atclink&userType=0&atcCode=N06A) | Venlafaxin |
| Other antidepressants | N06AX17 | Milnacipran |
| Other antidepressants | [N06AX18](https://www.fass.se/LIF/atcregister?1-1.-atccontainer-atcnavigatepanel4-listcontainer-atccodelistview-17-atclink&userType=0&atcCode=N06A) | Reboxetin |
| Other antidepressants | N06AX19 | Gepiron |
| Other antidepressants | [N06AX21](https://www.fass.se/LIF/atcregister?1-1.-atccontainer-atcnavigatepanel4-listcontainer-atccodelistview-19-atclink&userType=0&atcCode=N06A) | Duloxetin |
| Other antidepressants | [N06AX22](https://www.fass.se/LIF/atcregister?1-1.-atccontainer-atcnavigatepanel4-listcontainer-atccodelistview-20-atclink&userType=0&atcCode=N06A) | Agomelatin |
| Other antidepressants | N06AX23 | Desvenlafaxin |
| Other antidepressants | N06AX24 | Vilazodon |
| Other antidepressants | [N06AX26](https://www.fass.se/LIF/atcregister?1-1.-atccontainer-atcnavigatepanel4-listcontainer-atccodelistview-24-atclink&userType=0&atcCode=N06A) | Vortioxetin |
| Other antidepressants | N06AX28 | Levomilnacipran |
| Other antidepressants | N06AX29 | Brexanolon |

Table S3: ICD-10-SE and ATC codes for migraine measurement

| **Migraine exposure measurement** | | |
| --- | --- | --- |
| **Diagnosis (ICD-10-SE) codes** | | |
| Migraine | G43.0 | Migraine without aura |
| Migraine | G43.1 | Migraine with aura |
| Migraine | G43.2 | Status migrainosus |
| Migraine | G43.3 | Complicated migraine |
| Migraine | G43.8 | Other specified migraines |
| Migraine | G43.9 | Migraine, unspecified |
| **Prescription (ATC) codes** | | |
| CGRP | N02CD01 | Erenumab |
| CGRP | N02CD02 | Galkanezumab |
| CGRP | N02CD03 | Fremanezumab |
| CGRP | N02CD05 | Eptinezumab |
| CGRP | N02CD06 | Rimegepant |
| GGRP | N02CD07 | Atogepant |
| Triptan | N02CC01 | Sumatriptan |
| Triptan | N02CC02 | Naratriptan |
| Triptan | N02CC03 | Zolmitriptan |
| Triptan | N02CC04 | Rizatriptan |
| Triptan | N02CC05 | Almotriptan |
| Triptan | N02CC06 | Eletriptan |

Figure S1: Log minus log survival plot of depression or anxiety through end-2023 in the matched cohort

**
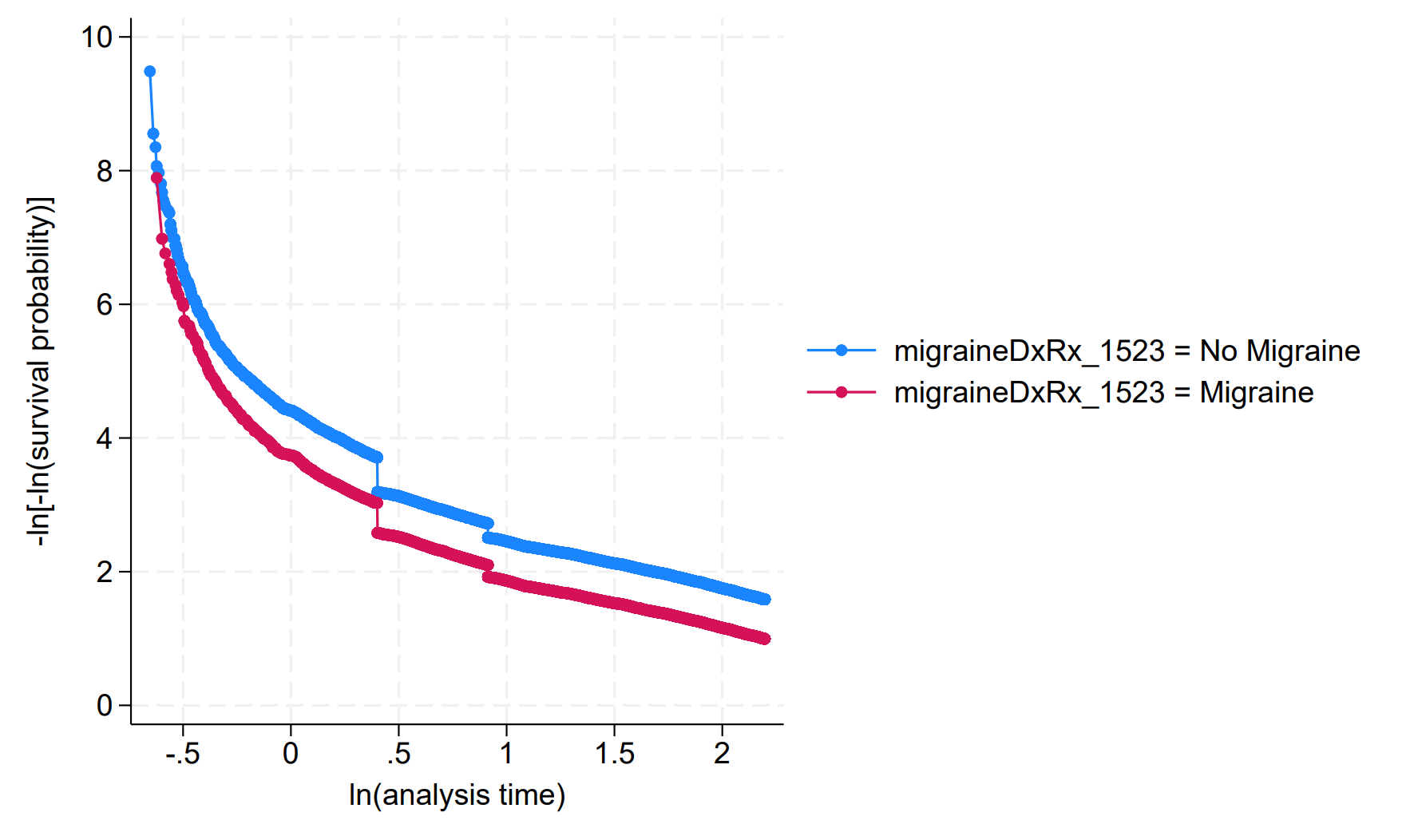
**

No Migraine

Migraine

Figure S2: Log minus log survival plot of depression or anxiety through end-2023 in the sibling cohort

**
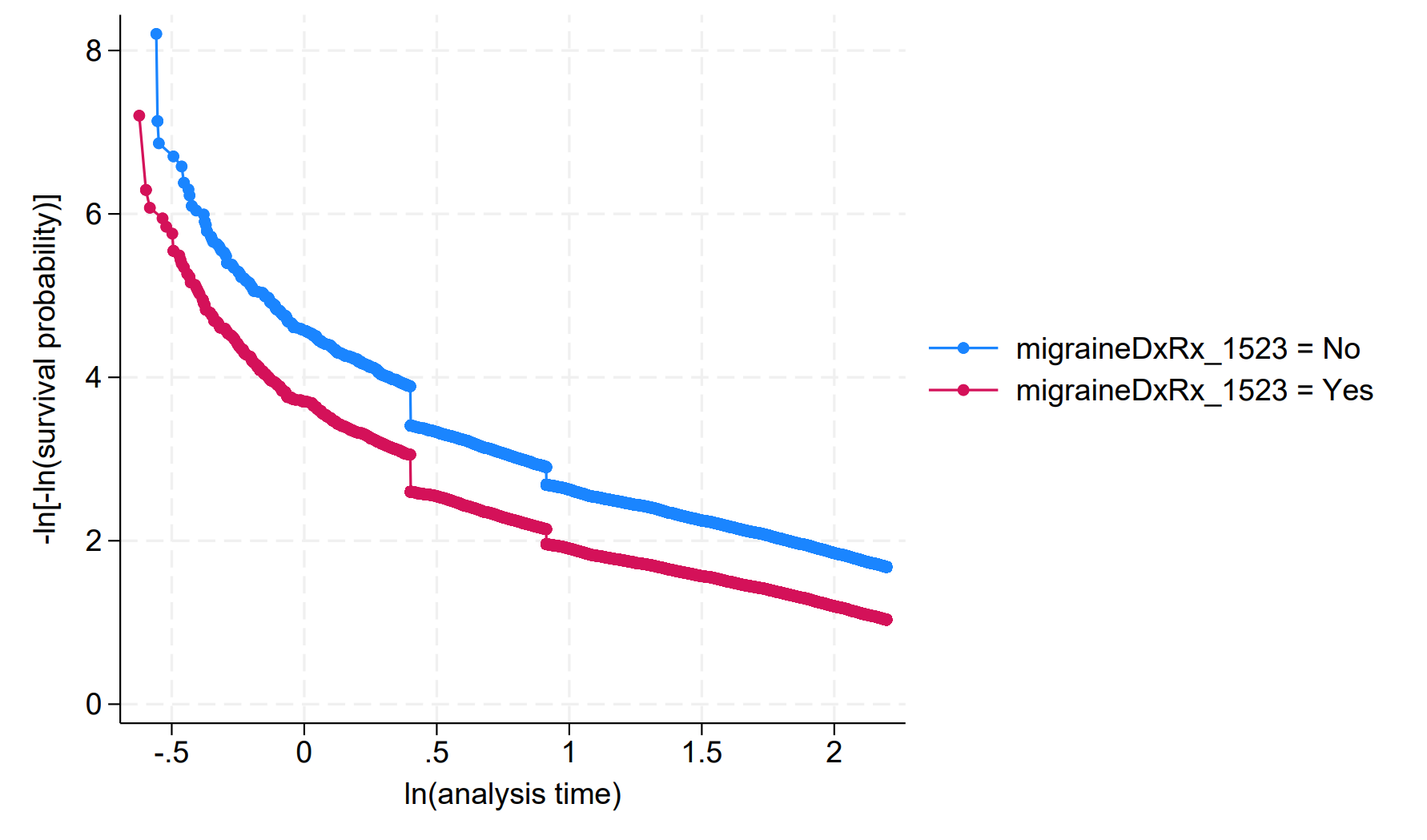
**

No Migraine

Migraine

Table S4: Sensitivity analysis of the association between migraine and diagnosed depression or anxiety through end-2023 in the matched cohort (excluding cases based on prescription treatment alone)

|  | **Persons entering follow-up** | **Diagnosed depression or anxiety** | **Person-years at risk** | **Rate of diagnosed depression or anxiety** | **Hazard ratios (95% CI)** | | | |
| --- | --- | --- | --- | --- | --- | --- | --- | --- |
|  | N | N | N | per 1,000 person-years | Crude | p-value | Adjusted | p-value |
| **Total matched population** | **1 490 372** | **83 418** | **7 565 649** | **10.7 (10.6 - 10.7)** |  |  |  |  |
| Migraine | 148 214 | 12 731 | 717 546 | 17.7 (17.4 - 18.1) | 1.73 (1.70 - 1.76) | <0.001 | 1.73 (1.70 - 1.76) | <0.001 |
| No migraine | 1 342 158 | 70 687 | 6 848 102 | 10.3 (10.3 - 10.4) | 1.00 |  | 1.00 |  |
| **Small-area deprivation** |  |  |  |  |  |  |  |  |
| Very high deprivation | 355 543 | 21 203 | 1 820 393 | 11.6 (11.5 - 11.8) | 1.08 (1.06 - 1.11) | <0.001 | 1.13 (1.10 - 1.15) | <0.001 |
| High deprivation | 369 410 | 21 137 | 1 866 730 | 11.3 (11.2 - 11.5) | 1.08 (1.05 - 1.10) | <0.001 | 1.11 (1.09 - 1.13) | <0.001 |
| Low deprivation | 378 978 | 20 749 | 1 907 965 | 10.9 (10.7 - 11.0) | 1.05 (1.03 - 1.07) | <0.001 | 1.07 (1.05 - 1.09) | <0.001 |
| Very low deprivation | 386 441 | 20 329 | 1 970 561 | 10.3 (10.2 - 10.5) | 1.00 |  | 1.00 |  |
| **Area of residence** |  |  |  |  |  |  |  |  |
| Urban | 1 152 714 | 65 521 | 5 829 660 | 11.2 (11.2 - 11.3) | 1.10 (1.07 - 1.12) | <0.001 | 1.13 (1.10 - 1.15) | <0.001 |
| Peri-urban | 123 834 | 6 887 | 637 605 | 10.8 (10.5 - 11.1) | 1.06 (1.03 - 1.10) | <0.001 | 1.07 (1.04 - 1.11) | <0.001 |
| Rural | 213 824 | 11 010 | 1 098 384 | 10.0 (9.8 - 10.2) | 1.00 |  | 1.00 |  |
| **Birthplace** |  |  |  |  |  |  |  |  |
| Nordic outside Sweden | 17 731 | 970 | 94 230 | 10.3 (9.7 - 11.0) | 1.05 (0.98 - 1.12) | 0.181 | 1.04 (0.97 - 1.11) | 0.284 |
| EU28 outside Nordic | 50 890 | 2 458 | 261 812 | 9.4 (9.0 - 9.8) | 0.90 (0.87 - 0.94) | <0.001 | 0.90 (0.86 - 0.94) | <0.001 |
| Europe outside EU28 | 43 896 | 2 273 | 225 108 | 10.1 (9.7 - 10.5) | 1.00 (0.95 - 1.04) | 0.827 | 0.96 (0.91 - 1.00) | 0.047 |
| Other birthplace | 179 779 | 8 749 | 916 316 | 9.5 (9.4 - 9.8) | 0.89 (0.87 - 0.91) | <0.001 | 0.86 (0.84 - 0.88) | <0.001 |
| Sweden | 1 198 076 | 68 968 | 6 068 184 | 11.4 (11.3 - 11.5) | 1.00 |  | 1.00 |  |

Note: Crude hazard ratios were estimated using stratified Cox regression models with strata of matched sets of exposed to unexposed (1:10) by sex and five-year age-bands without further adjustment. Adjusted hazard ratios were estimated with adjustment for age (continuous), sex, small-area deprivation, area of residence, and birthplace.

Table S5: Sensitivity analysis of the association between migraine and depression or anxiety through end-2023 in the matched cohort (excluding primary healthcare data)

|  | **Persons entering follow-up** | **Depression or anxiety episodes** | **Person-years at risk** | **Rate of depression or anxiety episodes** | **Hazard ratios (95% CI)** | | | |
| --- | --- | --- | --- | --- | --- | --- | --- | --- |
|  | N | N | N | per 1,000 person-years | Crude | p-value | Adjusted | p-value |
| **Total matched population** | **1 563 945** | **134 386** | **7 953 963** | **16.9 (16.8 - 17.0)** |  |  |  |  |
| Migraine | 123 201 | 16 851 | 550 383 | 30.6 (30.2 - 31.1) | 1.96 (1.93 - 2.00) | <0.001 | 1.96 (1.93 - 1.99) | <0.001 |
| No migraine | 1 440 744 | 117 535 | 7 403 580 | 15.9 (15.8 - 16.0) | 1.00 |  | 1.00 |  |
| **Small-area deprivation** |  |  |  |  |  |  |  |  |
| Very high deprivation | 374 051 | 33 413 | 1 919 872 | 17.4 (17.2 - 17.6) | 1.06 (1.05 - 1.08) | <0.001 | 1.09 (1.08 - 1.11) | <0.001 |
| High deprivation | 388 241 | 34 819 | 1 963 812 | 17.7 (17.5 - 17.9) | 1.11 (1.09 - 1.12) | <0.001 | 1.11 (1.09 - 1.12) | <0.001 |
| Low deprivation | 398 065 | 33 572 | 2 009 024 | 16.7 (16.5 - 16.9) | 1.05 (1.03 - 1.06) | <0.001 | 1.04 (1.03 - 1.06) | <0.001 |
| Very low deprivation | 403 588 | 32 582 | 2 061 256 | 15.8 (15.6 - 16.0) | 1.00 |  | 1.00 |  |
| **Area of residence** |  |  |  |  |  |  |  |  |
| Urban | 1 212 405 | 103 326 | 6 151 382 | 16.8 (16.7 - 16.9) | 0.96 (0.94 - 0.97) | <0.001 | 0.99 (0.97 - 1.01) | 0.194 |
| Peri-urban | 129 452 | 11 686 | 665 238 | 17.6 (17.3 - 17.9) | 1.02 (0.99 - 1.04) | 0.191 | 1.02 (1.00 - 1.05) | 0.029 |
| Rural | 222 088 | 19 374 | 1 137 344 | 17.0 (16.8 - 17.3) | 1.00 |  | 1.00 |  |
| **Birthplace** |  |  |  |  |  |  |  |  |
| Nordic outside Sweden | 18 709 | 1 443 | 99 946 | 14.4 (13.7 - 15.2) | 0.99 (0.94 - 1.05) | 0.798 | 0.99 (0.94 - 1.05) | 0.749 |
| EU28 outside Nordic | 53 034 | 4 269 | 272 488 | 15.7 (15.2 - 16.1) | 0.98 (0.95 - 1.01) | 0.207 | 0.98 (0.95 - 1.01) | 0.217 |
| Europe outside EU28 | 46 221 | 4 011 | 236 949 | 16.9 (16.4 - 17.5) | 1.06 (1.03 - 1.10) | 0.001 | 1.04 (1.01 - 1.08) | 0.011 |
| Other birthplace | 186 896 | 13 693 | 954 486 | 14.3 (14.1 - 14.6) | 0.86 (0.85 - 0.88) | <0.001 | 0.85 (0.84 - 0.87) | <0.001 |
| Sweden | 1 259 085 | 110 970 | 6 390 094 | 17.4 (17.3 - 17.5) | 1.00 |  | 1.00 |  |

Note: Crude hazard ratios were estimated using stratified Cox regression models with strata of matched sets of exposed to unexposed (1:10) by sex and five-year age-bands without further adjustment. Adjusted hazard ratios were estimated with adjustment for age (continuous), sex, small-area deprivation, area of residence, and birthplace.
